# Evolutionary Adaptability Coupled with Computation-Driven Engineering for Thermostability Enhancement of a Deoxynivalenol-Detoxifying Fusion Enzyme

**DOI:** 10.3390/ijms27177773

**Published:** 2026-08-30

**Authors:** Yiting Pan, Hao Zhu, Qingwei Jiang, Bin Ma, Changhe Chen, Fengxia Lu, Huibing Chi, Ping Zhu

**Affiliations:** College of Food Science and Technology, Nanjing Agricultural University, Nanjing 210095, China

**Keywords:** deoxynivalenol, fusion enzyme, thermostability, molecular dynamics simulation

## Abstract

Deoxynivalenol (DON), a trichothecene mycotoxin commonly found in cereal grains and their derived products, poses significant risks to human and animal health. In previous work, a fusion enzyme composed of the dehydrogenase DADH and the aldo-keto reductase AKR13B3 was engineered to convert DON into the non-toxic 3-epi-DON in a single step. However, the poor thermal stability of this fusion enzyme limited its industrial application. In this study, EVcouplings and the GRAPE-WEB platform were utilized to identify key amino acid residues governing the thermal stability of the fusion enzyme AKR13B3–DADH. Through single-point mutation screening and the combination of beneficial mutation sites, a triple mutant M361L/T508Y/Y603F (M1) was obtained. The half-life of M1 at 50 °C reached about 500 min, representing a 16.4-fold increase compared with the wild type, while its catalytic activity increased by 2.7-fold. The apparent melting temperature increased by approximately 4 °C. Molecular dynamics simulations verified that the improved thermostability results from reduced conformational flexibility in key regions, enhanced structural packing, and a strengthened hydrogen bond network. These results demonstrate the successful development of a thermostable DON-detoxifying fusion enzyme and provide a practical basis for its industrial application.

## 1. Introduction

Deoxynivalenol (DON) is a trichothecene mycotoxin that contaminates cereal grains and their derivatives worldwide. It poses serious hazards to human and animal health [1]. Conventional physical and chemical detoxification methods often suffer from incomplete toxin removal, and they can generate hazardous by-products [2]. They also tend to impair the sensory and nutritional quality of grain and feed [3,4]. Biological detoxification, which operates through microbial adsorption or secreted enzymes, circumvents these limitations. It offers stringent substrate specificity, avoids secondary pollution, and achieves high catalytic efficiency. Enzymatic approaches, in particular, combine operational simplicity, reusability, and a favorable safety profile [5].

Current research on biological DON detoxification focuses primarily on the C3 hydroxyl, and the C12–C13 epoxide [6]. The C3 epimerization pathway is of particular interest. It proceeds through a two-step transformation. The first step oxidizes DON to the intermediate 3-keto-DON, whose toxicity is several dozen-fold lower than that of the parent compound. The second step reduces this intermediate to the terminal product 3-epi-DON, which exhibits further attenuated toxicity [7]. In a previous work, the *Devosia* strain A6-243, capable of completely degrading DON, was isolated from soil. The enzymes responsible for this activity, DADH and AKR13B3, were subsequently identified from this strain. Because the native metabolic pathway involves two separate enzymes, a fusion enzyme AKR13B3–DADH was constructed in a previous work to enable the one-step conversion of DON into 3-epi-DON. The fusion enzyme simplifies the reaction workflow and eliminates the need for isolation of the toxic intermediate 3-keto-DON, thereby offering advantages over the two-enzyme system [7,8]. Despite this advance, the fusion enzyme suffers from poor thermostability, a shortcoming that limits its industrial utility. Feed pelleting is the predominant processing method in the feed industry, and it is known to improve animal growth performance and feed conversion efficiency. However, industrial pelleting typically operates between 65 and 80 °C [9,10]. This temperature range imposes stringent thermal demands on any enzyme intended for in-feed application. To date, no studies have reported protein engineering to improve the thermostability of DON-detoxifying fusion enzymes.

Recent progress in computation-guided protein engineering has opened new routes to address such limitations. Within a semi-rational design framework, tools including HotSpot Wizard, PoPMuSiC, FoldX, and Rosetta_ddg now allow the efficient identification of stabilizing mutations [11,12]. Peccati et al. developed a novel computational pipeline that integrates global conformational sampling with energy predictions based on AlphaFold and Rosetta to quantitatively predict the thermostability changes of acyltransferase LovD and lipase LipA during laboratory evolution [13,14,15]. Du et al. proposed a computer-assisted rational design strategy combining deep learning with multiple energy function methods to improve the thermostability of ulvan lyase. This approach employed ColabFold for structure prediction and utilized FoldX, Rosetta, and Schrödinger to screen for mutants with enhanced thermostability [16]. Zhang et al. applied machine learning to design combinatorial mutations for pectate lyase PMGL-Ba from *Bacillus* licheniformis, aiming to improve its thermostability [17]. Sun et al. developed a method termed “GReedy Accumulated Strategy for Protein Engineering” (GRAPE) to enhance enzyme stability for various applications. This method combines advanced computational approaches with a unique clustering and greedy accumulation strategy to efficiently explore epistatic effects with minimal experimental effort [18]. These computational tools provide feasible strategies for the thermal stability of DON-detoxifying fusion enzymes.

In this study, a semi-rational design strategy was employed to improve the thermostability of the AKR13B3–DADH fusion enzyme by co-evolutionary analysis and the GRAPE-WEB platform. Engineered mutants with greatly improved thermostability were successfully obtained. MD simulation and structural analysis were finally applied for investigating the improved resistant mechanism of AKR13B3–DADH to provide valuable information for further thermostability modification of other DON-detoxifying enzymes.

## 2. Results and Discussion

### 2.1. Identification of Key Residues Affecting the Thermostability of DON-Detoxifying Fusion Enzyme AKR13B3–DADH

**Figure 1:**
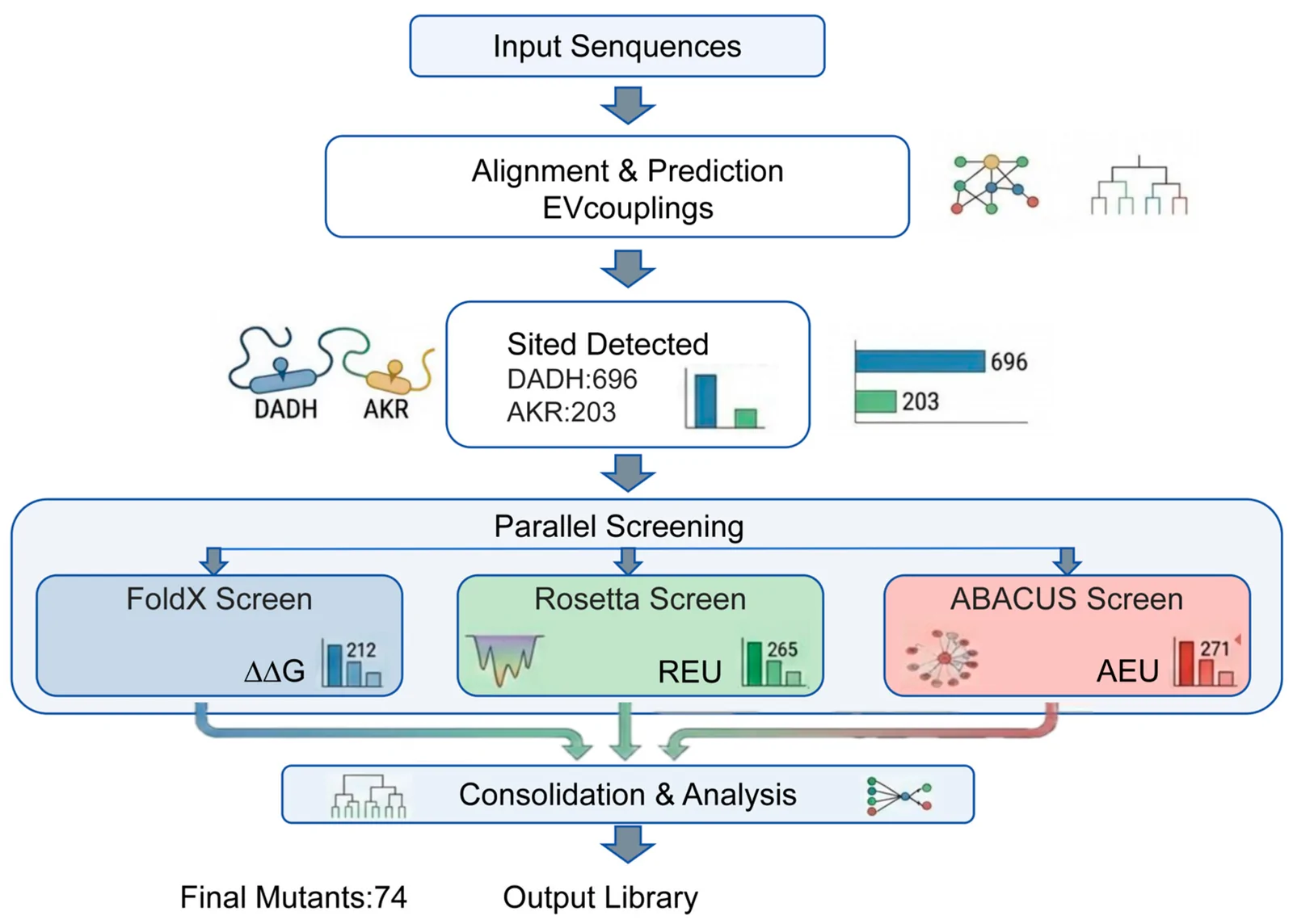
Screening workflow of the semi-rational design strategy for thermostability engineering of the fusion enzyme.

In this study, a semi-rational protein engineering strategy integrating co-evolutionary analysis and multiple computational scoring algorithms was adopted to screen stabilizing mutants of the fusion enzyme AKR13B3–DADH (Figure 1).

First, co-evolutionary coupling analysis was carried out to identify amino acid residues with the potential to improve enzyme thermostability. Using the EVcouplings software (https://v2.evcouplings.org/ (accessed on 25 March 2025)), a multiple sequence alignment of homologous sequences of the fusion enzyme was constructed. Residues that were fully conserved across the sequence alignment were eliminated from candidate mutation sites, as these residues were indispensable for maintaining protein folding and catalytic activity to prevent structural damage. In contrast, non-conserved residues with robust co-evolutionary coupling signals were selected as priority mutagenesis targets. Co-evolutionary coupling analysis was performed using the EVcouplings software with default parameters. The sequences of the DADH and AKR13B3 domains within the fusion enzyme were analyzed separately. Input sequences were obtained from our in-house laboratory collection, and the default sequence database provided by EVcouplings was used for multiple sequence alignment. The DADH domain was aligned against 8998 homologous sequences, while the AKR13B3 domain was aligned against 89,276 homologous sequences. The recommended output results at a bitscore of 0.5 were used for both domains. The result quality was 10 for both domains, which is the highest possible value in EVcouplings, indicating reliable analytical confidence. Candidate sites for mutagenesis were subsequently identified based on these results [4,19]. To ensure prediction reliability, an independent score threshold of 1 was applied to filter out low-aconfidence predictions. This analysis yielded 696 and 203 candidate mutation sites from the DADH and AKR13B3 domains, respectively, establishing a focused mutational landscape for subsequent computational design. The substantial difference in candidate numbers between the two domains may reflect variations in domain size, evolutionary conservation patterns, or their differential contributions to the overall structural stability of the fusion enzyme. By deliberately excluding absolutely conserved residues and enforcing a stringent confidence threshold, this strategy enriched for evolutionarily tolerated yet potentially stabilizing mutations, which likely contributed to the high hit rate subsequently observed during experimental screening of the 74-membered single-point mutant library (Figures S3 and S4).

Following the co-evolutionary screening, the full-length sequence of the fusion enzyme was imported into GRAPE-WEB, where multiple integrated computational scoring algorithms were applied to perform mutant design and stability assessment of the fusion enzyme. By evaluating changes in mutant thermostability from multiple dimensions based on their distinct energy functions and conformational sampling strategies, these algorithms collectively helped mitigate the risk of false positives arising from the prediction bias of any single algorithm [11]. To improve screening specificity, the energy cutoff thresholds for the three mainstream algorithms were set as follows: FoldX, −1.5 kcal/mol; Rosetta, −1.0 REU; and ABACUS, −2.5 AEU. Independent screening with each algorithm yielded 271, 265, and 212 candidate mutants, respectively.

Finally, all candidate mutants identified by the three algorithms were integrated and subjected to cluster analysis, generating a compact mutant library containing only 74 mutants. This library was designed to enable the synergistic optimization of the catalytic activity and thermostability of the fusion enzyme with a relatively modest experimental throughput, thereby providing a high-quality candidate pool for subsequent experimental validation.

### 2.2. Impact of Mutations on the Catalytic Activity and Thermostability of DON-Detoxifying Fusion Enzyme AKR13B3–DADH

**Figure 2:**
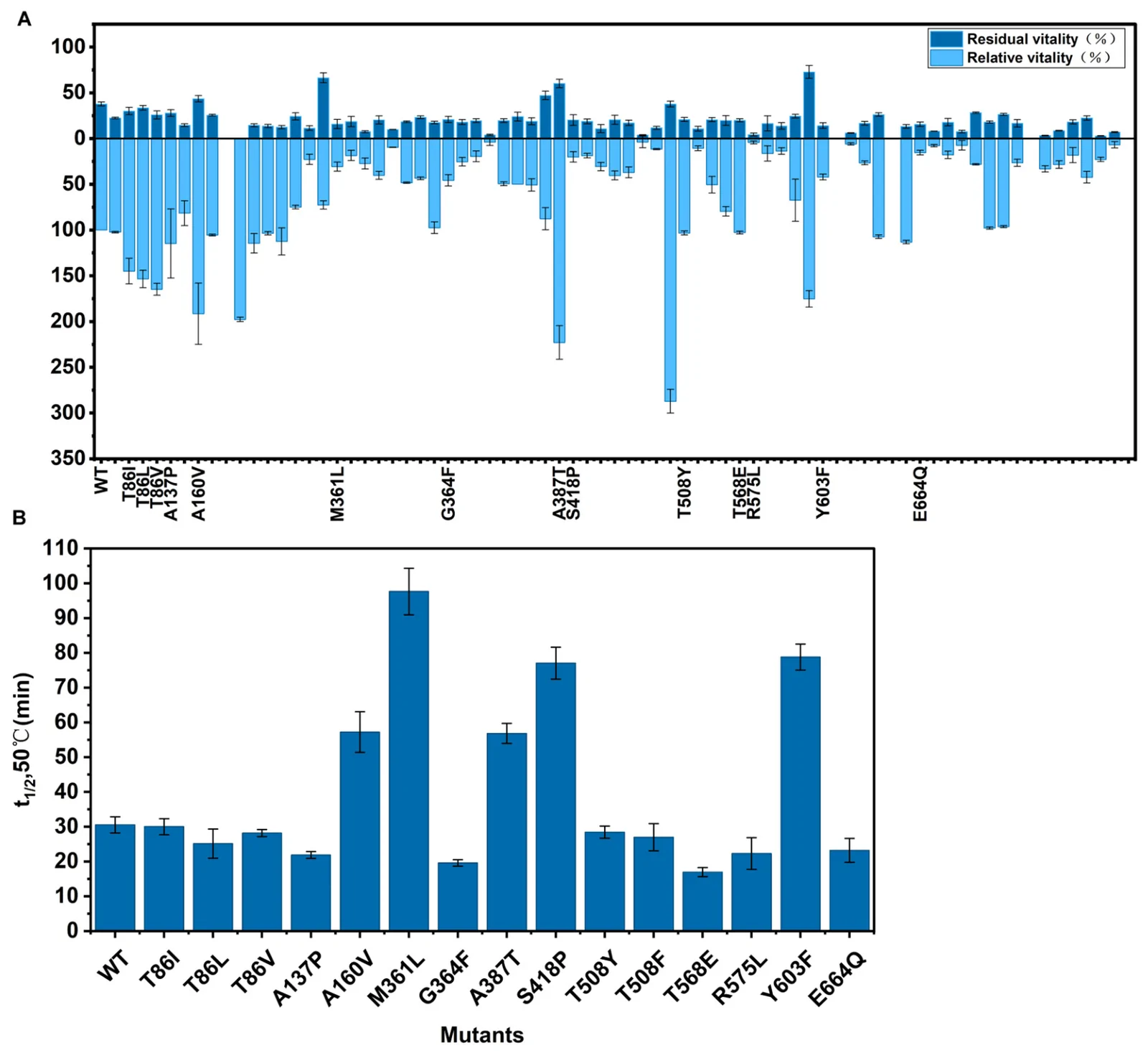
(**A**) Catalytic activity and residual activity after heat treatment of the single-point mutants. (**B**) Half-life at 50 °C of the 15 single-point mutants subjected to secondary screening.

To evaluate the thermostability of the predicted mutants, all 74 single-point mutants were incubated at 50 °C, after which their residual activities were measured as an initial assessment of thermostability. Their catalytic activities were also preliminarily screened, with the activity of the WT set as 100% (Figure 2A and Figure S1). Six mutants exhibited significantly improved residual activity compared with the WT, and nine mutants showed markedly enhanced catalytic activity without a substantial loss of residual activity; the remaining mutants displayed varying degrees of decline in both catalytic activity and thermostability. Notably, the single-point mutants S174L, G221R, S612V, E664V, and D758L completely lost their ability to degrade DON, suggesting that these mutation sites are likely key residues essential for maintaining an active conformation.

To further identify single-point mutants with enhanced thermostability, the 15 mutants obtained from the initial screening were subjected to half-life (*t*_1/2_) measurement at 50 °C (Figure 2B). Among them, mutants T86I, T86L, T86V, T508Y, and T508F showed no significant improvement in *t*_1/2_ at 50 °C compared with WT, whereas mutants A137P, G364F, T568E, R575L, and E664Q exhibited a decrease in half-life to varying degrees. After this secondary screening, six single-point mutants A160V, M361L, A387T, S418P, T508Y, and Y603F were found to display improved catalytic activity and thermostability to different extents (Table S2). Specifically, the catalytic activity of mutant T508Y reached 287.2% of that of the WT, and the *t*_1/2_ of mutant M361L at 50 °C was 97.6 min, representing a 3.2-fold increase over that of the WT.

Among the single-point mutations, M361L contributed most significantly to thermostability, conferring a 3.2-fold increase in *t*_1/2_ at the single-point level. This mutation likely enhanced hydrophobic packing within the protein core, a well-recognized mechanism by which methionine-to-leucine substitutions stabilize proteins [20,21,22]. In contrast, mutants such as S174L, G221R, S612V, E664V, and D758L completely lost their DON-degrading ability, indicating that these residues, although perhaps not located within the annotated active site, are essential for maintaining an active conformation. It is hypothesized that these sites play critical roles in domain–domain interactions, cofactor (NADPH/PQQ) binding, or the structural integrity of substrate-binding loops within the fusion enzyme architecture [23]. The identification of these residues provides valuable targets for future structure–function studies.

The six single-point mutants exhibiting varying degrees of improvement in both thermostability and catalytic activity were further subjected to a combinatorial strategy to construct combined mutants, which were subsequently screened for enhanced thermostability.

**Figure 3:**
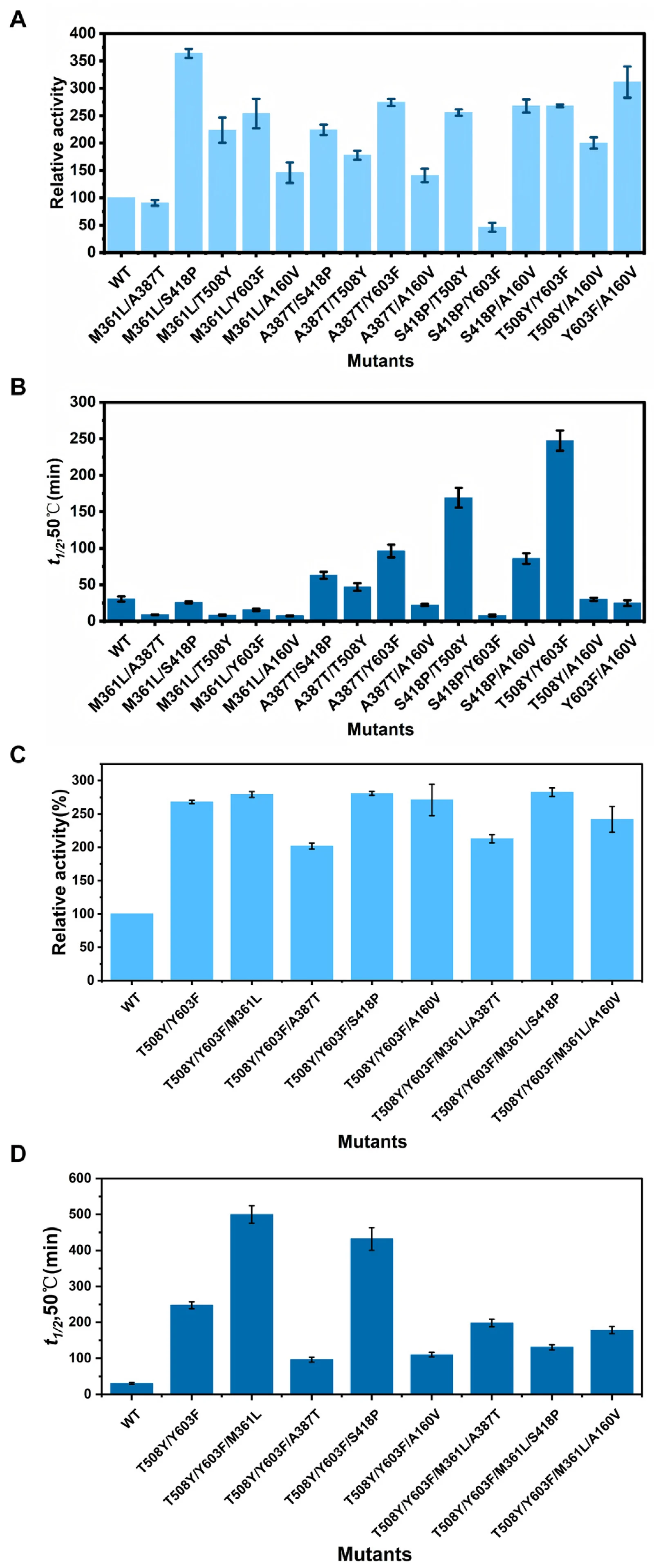
(**A**) Catalytic activities of the double-point combinatorial mutants. (**B**) Half-lives of the double-point combinatorial mutants. (**C**) Catalytic activity of the triple-point and quadruple-point combinatorial mutants. (**D**) Half-life of the triple-point and quadruple-point combinatorial mutants.

The six single-point mutants obtained from the screening—A160V, M361L, A387T, S418P, T508Y, and Y603F—were randomly paired to construct 15 double-point mutants (Table S3), which were then screened for thermostability (Figure 3). Among these, the double-point mutant T508Y/Y603F exhibited a catalytic activity 2.7 times that of the WT and a *t*_1/2_ at 50 °C of 247.55 min, representing an 8-fold increase relative to the WT. After heat treatment at 50 °C for 3 h, this double-point mutant retained more than 50% of its residual activity, demonstrating markedly superior thermostability compared to the WT.

Based on the double-point mutant T508Y/Y603F, which exhibited the most significant improvement in thermostability, the remaining beneficial single-point mutations were combined to construct triple-point combinatorial mutants (Table S4). These mutants were then subjected to thermostability screening (Figure 3A,B). The triple-point mutants were incubated at 50 °C for various time intervals to further evaluate their thermostability. As shown in Figure 3A, the catalytic activities of all triple-point mutants were improved to varying degrees compared with those of the WT and the double-point mutant. Relative to the parental double-point mutant T508Y/Y603F, the triple-point mutants M1 (T508Y/Y603F/M361L) and T508Y/Y603F/S418P exhibited significantly enhanced thermostability, whereas the half-lives of the triple-point mutants T508Y/Y603F/A387T and T508Y/Y603F/A160V were not improved, and their thermostability was even compromised. Ultimately, the triple-point mutant M1 (M361L/T508Y/Y603F) retained more than 50% of its residual activity after incubation at 50 °C for 420 min, and its catalytic activity was 2.71-fold higher than that of the WT (Figure 3C,D), demonstrating superior thermostability compared with the wild-type enzyme.

Further combinatorial extension to quadruple-point mutants using M1 as the template did not yield any variants with thermostability surpassing that of M1 (Figure 3A,B). This result suggested that M1 might represent a local fitness optimum within the explored sequence space, where additional mutations disrupt the favorable interaction network established by the three beneficial substitutions [24,25].

Through the combinatorial strategy, a triple-point mutant, designated M1, was ultimately obtained. Its half-life at 50 °C reached about 500 min, representing a 16.4-fold increase relative to that of the WT. The properties of the mutant were further evaluated under various biochemical characterization conditions.

### 2.3. Enzymatic Property Characterization of the Wild-Type and Mutant Enzymes

**Figure 4:**
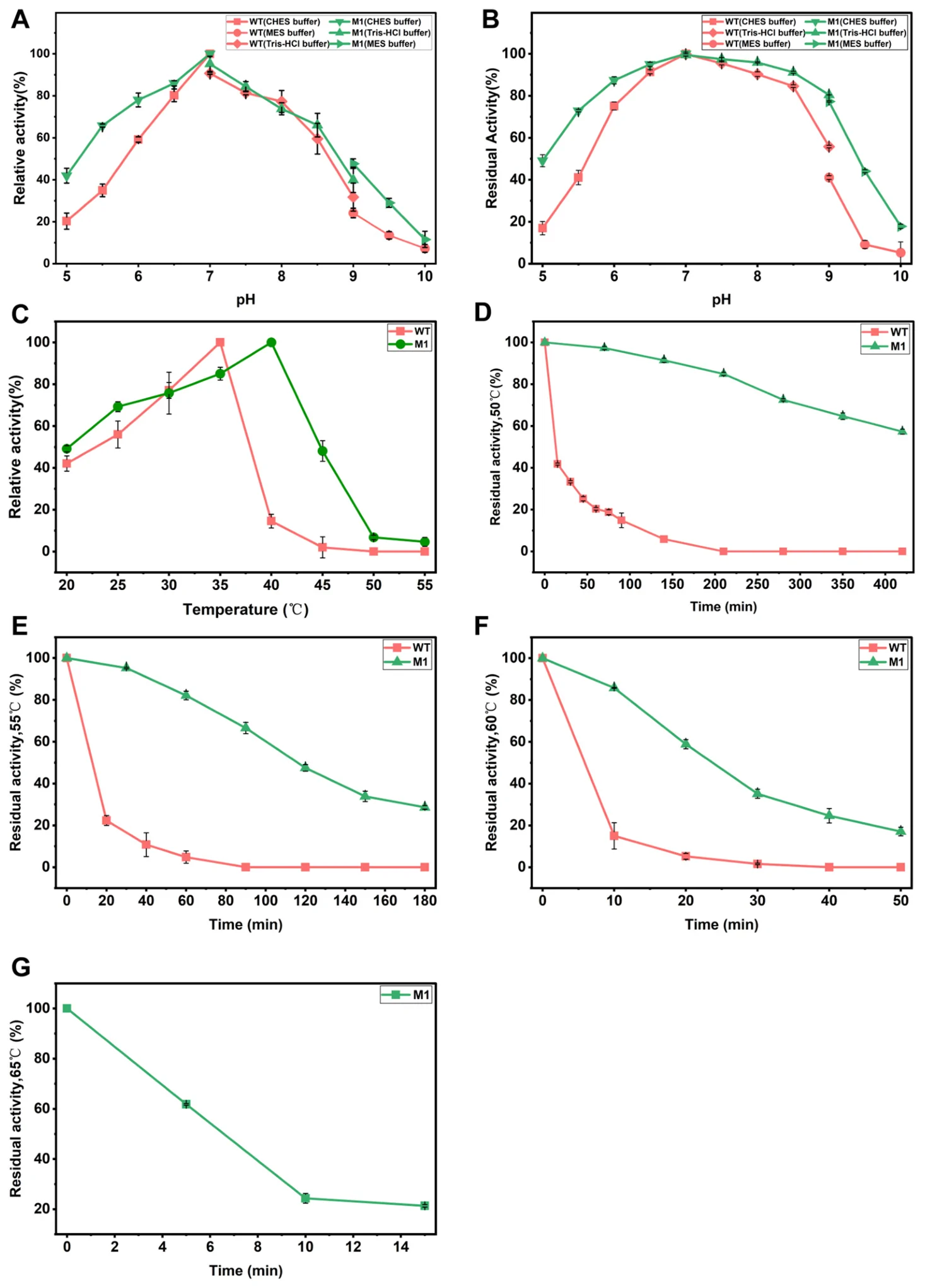
(**A**) Optimal pH curves of WT and M1. (**B**) pH stability curves of WT and M1. (**C**) Optimal temperature curves of WT and M1. (**D**) Thermostability curves of WT and M1 at 50 °C. (**E**) Thermostability curves of WT and M1 at 55 °C. (**F**) Thermostability curves of WT and M1 at 60 °C. (**G**) Thermostability curve of M1 at 65 °C.

The effect of pH on the WT and M1 enzymes is shown in Figure 4A. The optimal pH for both WT and M1 was 7 (Figure 4B). The WT retained more than 50% of its relative activity between pH 6 and 8.5, whereas M1 maintained over 50% relative activity across a broader range of pH 5.5 to 8.5. Furthermore, M1 exhibited higher overall activity than WT across the entire pH range of 5 to 10 (Figure 4B), demonstrating that its pH stability was improved after mutation, while the overall pH activity profile remained broadly consistent with that of WT. Both enzymes were therefore best suited to function in neutral to slightly alkaline environments.

The effect of reaction temperature on WT and the M1 mutant is illustrated in Figure 4C. The optimal reaction temperature of WT was 35 °C, whereas that of M1 shifted upward to 40 °C. At 40 °C, WT retained only 14.54% of its residual activity, and it was almost completely inactivated at 45 °C. In contrast, M1 still retained 48% of its residual activity at 45 °C. The optimal temperature profiles clearly indicated that M1 exhibits significantly enhanced thermostability compared with WT and is better adapted to elevated reaction temperatures.

The thermostability of the triple-point mutant was further characterized in detail. As shown in Figure 4E–G, the *t*_1/2_ of WT and M1 were determined at 55 °C, 60 °C, and 65 °C. At 55 °C, WT retained about 20% of its activity after 20 min of incubation, with a *t*_1/2_ of 14 min. At 60 °C, its residual activity dropped to about 15% after 10 min. At 65 °C, WT was rapidly inactivated within 3 min. In stark contrast, M1 retained about 50% of its residual activity after 120 min at 55 °C, more than 20% after 40 min at 60 °C, and more than 60% after 5 min of heat treatment at 65 °C. This comprehensive thermostability characterization at elevated temperatures demonstrates that the triple-point mutant M1 possesses vastly superior thermostability compared with the wild-type enzyme, underscoring its potential for future industrial applications.

Enzymatic characterization of M1 revealed broadened operational pH and temperature ranges without altering its inherent pH preference. M1 retained the same optimal pH as the WT (pH 7), yet maintained over 50% relative activity across a wider pH window (5.5–8.5, compared with 6–8.5 for the WT) and exhibited higher overall activity than the WT from pH 5 to 10 (Figure 4A,B), suggesting that its enhanced pH robustness may stem from global stabilization of the protein scaffold. The optimal reaction temperature of M1 shifted from 35 °C to 40 °C, and the mutant retained significant activity under conditions where the WT was nearly or completely inactivated (48% residual activity at 45 °C, whereas the WT was almost fully inactivated) (Figure 4C). At 55 °C, the *t*_1/2_ of M1 was far longer than that of the WT; at 65 °C, M1 retained over 60% residual activity after 5 min, whereas the WT was completely inactivated within 3 min (Figure 4E–G). Even after incubation at 65 °C for 10 min, M1 retained approximately 20% residual activity. This indicates that the mutations conferred functionally meaningful thermal tolerance under extreme heat stress, suggesting that M1 may be a promising candidate for further optimization toward potential industrial applications.

The apparent melting temperature (*T_m_*) is an important indicator for evaluating thermostability. In this study, the *T_m_* values of the WT and the mutant M1 were determined by differential scanning fluorimetry (DSF). As shown in Table S5, the experimental results showed that the apparent melting temperature of the WT was 50.8 °C, whereas that of M1 was 54.9 °C, representing an increase of approximately 4 °C relative to the WT.

As a key thermodynamic parameter for assessing enzyme thermostability, the apparent *T_m_* directly reflects the resistance of an enzyme molecule to thermal denaturation. Generally, a higher *T_m_* value indicates a stronger ability of the enzyme to maintain its native conformation under high-temperature conditions, and consequently, greater thermostability. In this study, the *T*_m_ value of the mutant M1 was markedly elevated compared to that of the WT, indicating that M1 possessed a greater capacity to retain catalytic activity in high-temperature environments and was better adapted to reactions under such conditions [26,27,28].

### 2.4. Molecular Dynamics Simulations Analysis of the Wild-Type and Mutant Enzymes

To elucidate the molecular mechanism underlying the enhanced thermostability of mutant M1, 100 ns molecular dynamics simulations were performed on the structures of the WT and the mutant M1.

**Figure 5:**
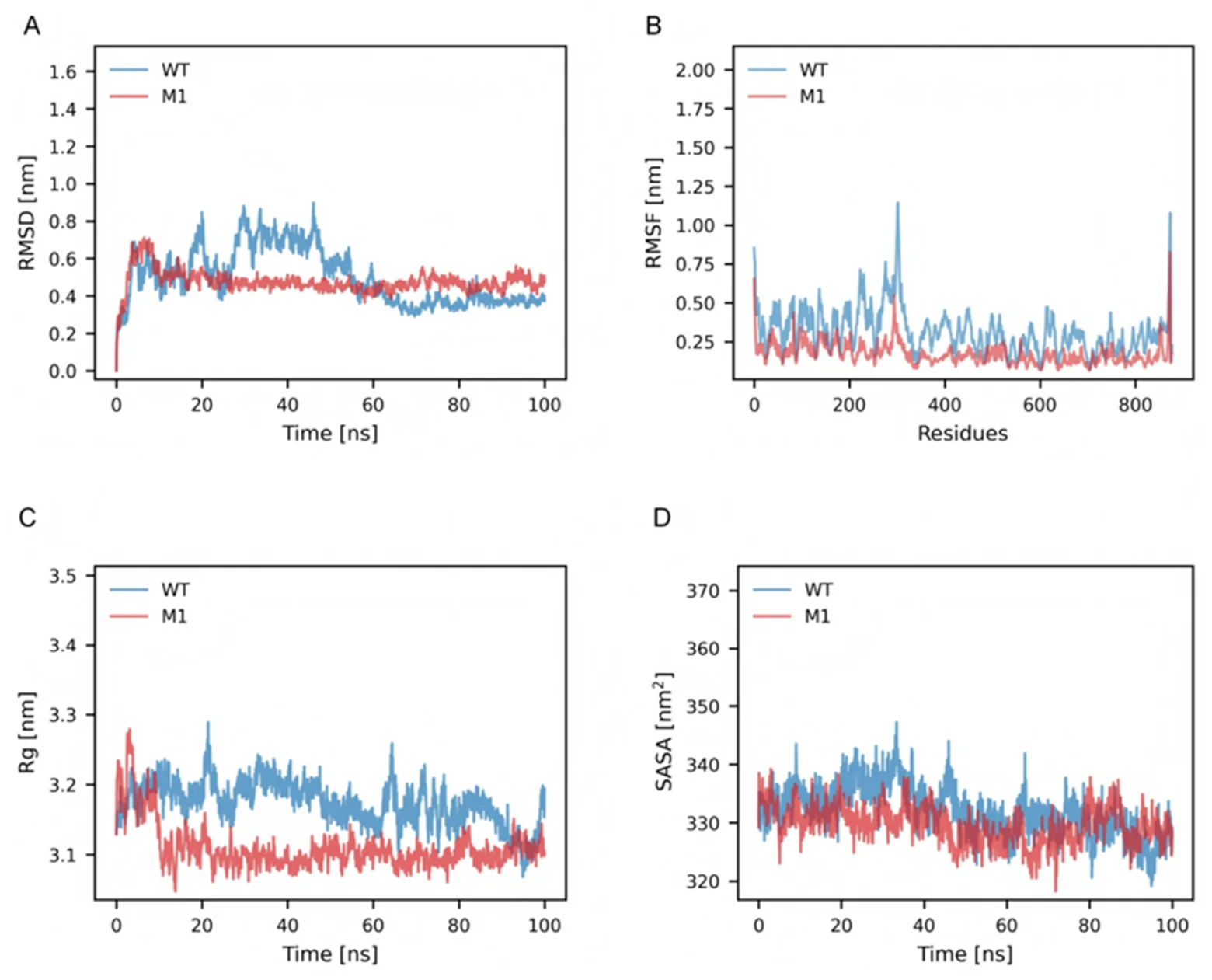
Molecular dynamics simulation results of the WT and M1. (**A**) Complex RMSD during MD simulation. (**B**) Complex RMSF during MD simulation. (**C**) Rg during MD simulation. (**D**) SASA during MD simulation as a function of simulation time.

Root-mean-square deviation (RMSD) analysis was conducted on the WT and M1 complexes. The RMSD of the complex was used to evaluate the overall conformational stability of the system throughout the simulation. As shown in Figure 5A, the RMSD of the WT system increased rapidly during the early stage of the simulation, indicating that the WT complex underwent pronounced overall conformational rearrangement under thermal motion. Although the RMSD subsequently declined gradually and stabilized, the substantial fluctuations observed during the intermediate stage reflected its relatively weak thermostability. In contrast, the M1 system reached a plateau relatively quickly after an initial increase to approximately 0.5–0.6 nm, with overall fluctuation amplitudes lower than those of the WT. These results indicate that the mutant attained thermal equilibrium more rapidly and was able to maintain a more stable complex conformation at the simulation temperature.

Root-mean-square fluctuation (RMSF) analysis (Figure 5B) revealed that M1 exhibited lower flexibility than the WT in most residue regions. In particular, within the residue ranges of approximately 50–300 and 400–800, M1 displayed generally smaller fluctuations, suggesting an overall increase in the rigidity of the mutant and a reduced response of local structures to thermal perturbations. The WT exhibited a pronounced peak near residue 300, with an RMSF value exceeding 1.0 nm, indicating strong local thermal motion or conformational instability in this region. Near the C-terminal end of the simulation, the WT also displayed relatively high fluctuations. In comparison, the peak intensities in these regions were markedly reduced in M1, suggesting that the T508Y/Y603F/M361L mutations may stabilize residues in key loop regions, inter-domain linkers, or substrate channel vicinities, thereby reducing thermally induced local disordered motions. This reduction in local flexibility represents a key dynamic feature underlying the enhanced thermostability of the mutant.

The radius of gyration (Rg) was used to characterize the overall compactness of the complex (Figure 5C). Throughout the simulation, the Rg of the WT complex was consistently higher than that of M1 and was accompanied by certain fluctuations, suggesting that the WT adopted a relatively looser conformation and may have experienced more pronounced thermally induced domain breathing motions or active pocket expansion. The lower and more stable Rg value of M1 indicates that the overall conformation of the mutant complex was more compact and less prone to significant expansion or loosening under thermal motion.

The solvent-accessible surface area (SASA) reflects the degree of surface exposure and conformational compactness of the complex (Figure 5D). The SASA values of both the WT and M1 complexes were primarily distributed within the range of approximately 325–340 nm^2^, indicating that neither system underwent substantial unfolding or large-scale conformational disruption during the simulation. However, the SASA of the WT exhibited a slight increase during the period of approximately 30–45 ns, reaching a maximum of nearly 345 nm^2^, suggesting an increase in the surface exposure of the complex during this phase, which may correspond to the intermediate-stage conformational fluctuations observed in the RMSD results of the WT. In contrast, the SASA of the M1 system was slightly lower overall and its fluctuations were relatively moderate, indicating a lower degree of surface exposure and a more compact structure of the mutant complex. The lower SASA and lower Rg values mutually corroborate each other, demonstrating that M1 is less susceptible to thermally induced structural loosening compared to the WT [27,29,30].

The hydrogen bond network is one of the key non-covalent interactions maintaining the thermostability of protein complexes (Figure S2). The results showed that the M1 system consistently formed 1–3 hydrogen bonds throughout the simulation, and in certain periods this number reached 4, whereas the WT system exhibited fewer hydrogen bonds overall with a sparser distribution. The more frequent and more stable hydrogen bond formation in M1 indicates that the mutations enhanced the polar interactions between the substrate/cofactor and the active pocket of the protein, thereby imposing stronger structural constraints on the complex under thermal fluctuations. Compared with the WT, the more stable hydrogen bond network in M1 contributed to maintaining the conformation of the active pocket and substrate orientation, thereby reducing conformational drift induced by thermal motion [31,32].

Collectively, the M1 mutant exhibited a more stable complex conformation, lower local protein flexibility, a more compact overall structure, more moderate changes in surface exposure, and more persistent hydrogen bond interactions compared to the WT. From a thermostability perspective, the WT displayed more pronounced fluctuations in RMSD, RMSF, Rg, and SASA, indicating that it is more susceptible to local softening, conformational rearrangement, and complex loosening under thermal motion [33,34]. In contrast, M1 enhanced the tolerance of the active pocket and the overall complex to thermal fluctuations by reducing flexibility in key regions, strengthening structural compactness, and stabilizing polar interactions. Overall, the M1 mutations rendered the complex more stable, more compact, and less prone to conformational drift under thermal motion, suggesting that its thermostability is superior to that of the WT [26,35].

**Figure 6:**
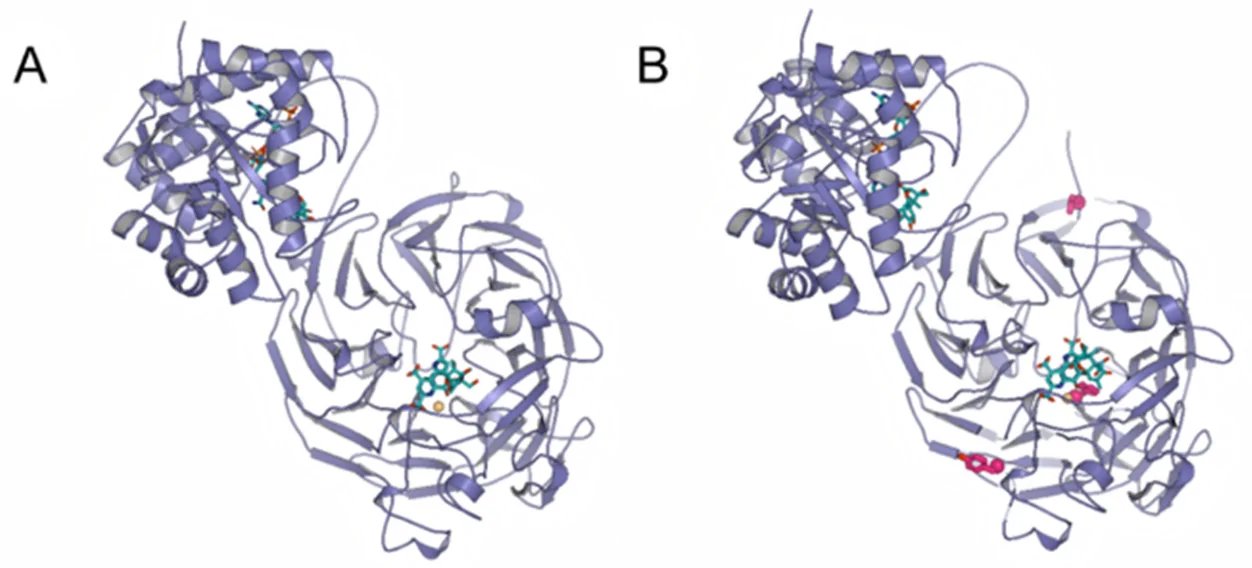
(**A**) Structural model of WT. (**B**) Structural model of M1.The red portions indicate the mutated residues.

To investigate the spatial arrangement of the mutation sites and their potential contributions to enhanced thermostability, three-dimensional structures of the wild-type AKR13B3–DADH fusion enzyme (WT) and the M1 mutant were predicted, and the docking conformations of the substrate within the modeled structures are presented (Figure 6A,B). The three mutated residues in M1 are all located within the DADH domain, with their side chains highlighted in the structural representations. Among them, M361L is positioned far from the active center (29.11 Å from the substrate), T508Y serves as a contact residue on the outer surface of the active-site pocket, and Y603F resides at the rim of the active pocket (8.36 Å). At the mechanistic level, M361L reduces side-chain conformational freedom and improves hydrophobic core packing; T508Y further reinforces local rigidity within the pre-existing aromatic environment formed by Y492 and F509; and Y603F optimizes local hydrophobicity. Although the single T508Y point mutant did not enhance the thermostability of the fusion enzyme (Figure 2B), its combination with Y603F resulted in a marked improvement in thermostability (Figure 3B), likely attributable to synergistic conformational optimization. Overall, the three mutations primarily modulate local packing and conformational rigidity [36,37]. The spatial distribution of these mutations may constitute a rational combination spanning the distal, intermediate, and proximal regions.

The distal mutation M361L likely promotes global stability by optimizing hydrophobic core packing within the DADH domain, an effect consistent with its predominant role in extending the half-life observed in single-point screening. The intermediate-range mutation T508Y, situated on the outer surface of the active-site pocket, may stabilize the local conformation by reinforcing the existing aromatic cluster formed by Y492 and F509, thereby rigidifying the enzyme structure without directly perturbing catalytic residues. The proximal mutation Y603F, located at the rim of the active pocket, may finely modulate the local hydrophobic microenvironment, thus contributing to the enhanced catalytic activity of M1. Importantly, the absence of direct mutations within the catalytic center suggests that the improved thermostability of M1 is achieved primarily through peripheral and distal stabilization effects, rather than through direct modification of the active-site chemical environment. This spatial architecture may explain how M1 simultaneously attains enhanced stability and improved catalytic efficiency, circumventing the stability–activity trade-off commonly encountered in enzyme engineering. Collectively, the distal-to-proximal spatial deployment of the three mutations likely represents a finely orchestrated structural solution that confers thermostability while maintaining or even improving catalytic function.

## 3. Materials and Methods

### 3.1. Chemicals, Strains, and Plasmids

DON and 3-keto-DON were purchased from TripleBond (Guelph, Ontario, Canada). Reduced nicotinamide adenine dinucleotide phosphate (NADPH) and pyrroloquinoline quinone (PQQ) were all obtained from Beyotime Biotechnology Co., Ltd. (Shanghai, China). Kanamycin and isopropyl-β-D-thiogalactopyranoside (IPTG) were purchased from Solarbio Science & Technology Co., Ltd. (Beijing, China). Protein molecular weight markers were purchased from Yeasen Biotechnology Co., Ltd. (Shanghai, China). All other chemical reagents used in this study were of analytical grade.

The plasmid for the DON-degrading fusion enzyme AKR13B3–DADH was previously constructed and stored in our laboratory (College of Food Science and Technology, Nanjing Agricultural University). *Escherichia coli* BL21 and the plasmid pET28a were maintained in our laboratory (College of Food Science and Technology, Nanjing Agricultural University).

### 3.2. Gene Cloning and Plasmid Construction

Using the wild-type plasmid as a template, target genes were amplified by polymerase chain reaction using the corresponding primer pairs (Table S1). Site-directed mutagenesis was carried out by whole-plasmid PCR with mutagenic primers. Full-length plasmids were obtained through PCR amplification. The PCR products were digested with DpnI and purified using a FastPure Gel DNA Extraction Mini Kit. The purified DNA was then transformed into competent Escherichia coli BL21 (DE3) cells. All mutants were verified by DNA sequencing to confirm correct plasmid construction for DADH expression.

### 3.3. Semi-Rational Design

We used EVcouplings to perform multiple sequence alignment of homologous sequences and to predict single-point mutations [19,33]. The predicted independence score served as the screening threshold. This analysis identified a large set of potential mutants in the DADH domain and a separate set in the AKR domain. We then employed the GRAPE-WEB platform to guide the design of fusion enzyme mutants. Three algorithms were applied to evaluate candidate mutations: FoldX, Rosetta, and ABACUS [18]. Mutations were filtered according to the appropriate energy cutoffs that were established for each algorithm, which produced a distinct candidate pool per method. We combined these pools and performed cluster analysis to construct a mutant library that balances catalytic activity and thermostability.

### 3.4. Expression and Purification of Enzymes

Plasmids carrying the respective enzyme genes were transformed into E. coli BL21 competent cells, which were then spread onto kanamycin-containing plates and incubated overnight at 37 °C. Single colonies were picked and inoculated into 30 mL of LB medium containing kanamycin (50 μg/mL) and cultured overnight at 37 °C with shaking at 200 rpm. Subsequently, 1 mL of the culture was transferred into 100 mL of fresh LB medium and cultivated at 37 °C and 200 rpm until the OD_600_ reached 0.6–0.8. IPTG was added to a final concentration of 0.2 mM, and protein expression was induced at 16 °C for 20 h with shaking at 200 rpm. Cells were harvested by centrifugation (8000 rpm, 3 min, 4 °C), and the cell pellet was resuspended in 6 mL of resuspension buffer (50 mM Tris, pH 7.5, 200 mM NaCl). The suspension was subjected to ultrasonication for 8 min, followed by centrifugation (9000 rpm, 15 min, 4 °C). The resulting supernatant was collected as the crude enzyme extract.

The crude enzyme extract was purified by affinity chromatography using a 10 mL HisTrap prepacked column. After sample loading, the column was washed with one column volume of wash buffer (50 mM Tris-HCl, pH 7.5, 200 mM NaCl, 50 mM imidazole) to remove non-specifically bound proteins, and the target protein was subsequently eluted with elution buffer (50 mM Tris-HCl, pH 7.5, 200 mM NaCl, 500 mM imidazole). Following overnight dialysis to remove imidazole, the protein concentration and enzyme activity were determined. The purity of the purified wild-type and mutant fusion enzymes was assessed by SDS-PAGE.

### 3.5. Enzyme Activity Assay

The thermostability of the WT and its mutants was evaluated by incubating the purified enzymes at a final concentration of 0.5 mg/mL in 50 mM Tris-HCl, 200 mM NaCl, pH 7.5. Incubations were carried out at specified temperatures (50, 55, 60, or 65 °C) for various time intervals. At predetermined time points, aliquots were withdrawn and immediately cooled on ice for 10 min to terminate thermal inactivation. Residual activity was then determined under standard assay conditions: the reaction mixture (100 μL) contained 50 mM Tris-HCl buffer (pH 7.5), 10 mM DON, 20 μM PQQ, and 1 mM Ca^2+^, at 37 °C for 90 min, unless otherwise stated. The activity of the untreated enzyme incubated on ice was defined as 100%. All measurements were performed in triplicate, and data are presented as mean ± standard deviation (SD). The mixture was filtered through a 0.22 μm organic membrane filter and separated on a C18 reversed-phase column. The mobile phase consisted of water (phase A) and methanol (phase B) at a 70:30 (*v*/*v*) ratio. The detection parameters were as follows: flow rate, 0.6 mL/min; detection wavelength, 218 nm; injection volume, 20 μL. We performed quantification using a standard curve that was generated from the reaction product.

### 3.6. Thermostability Evaluation

To characterize the half-life (*t*_1/2_) of the wild-type and mutant enzymes, the purified enzymes were incubated at 50 °C, and the residual enzyme activity was measured according to the method described in Section 3.5. The residual activity was expressed as the ratio of the activity after heat treatment to the initial activity. The *t*_1/2_ value was calculated using the following equation:

$$t_{1/2}\;=\;\ln2/\mathrm{ki}$$

The inactivation rate constant (ki) was determined from the first-order linear regression equation lnA = ki × t, with A representing residual activity and t representing incubation time.

### 3.7. Enzymatic Property Characterization

We determined the optimal reaction temperature by measuring the catalytic activities of the wild-type fusion enzyme and its mutants across a temperature range of 20 to 55 °C. Thermostability was evaluated by incubating the enzymes at 50 °C and measuring residual activity. We determined the optimal pH by assaying catalytic activity at 37 °C across a pH range of 5 to 10. To evaluate pH stability, we incubated the wild-type and its mutants in buffers of different pH values at 4 °C for 12 h and then measured residual activity. The buffers used were CHES (pH 5 to 7), Tris-HCl (pH 7 to 9), and MES (pH 9 to 10). All enzyme activities were assayed according to the method described in Section 3.5.

### 3.8. Determination of Apparent Melting Temperature (T_m_)

The *T_m_* value was derived from the resulting melting curve. The thermal unfolding of the wild-type and its mutants was monitored by differential scanning fluorimetry (DSF) using a QuantStudio 5 Real-Time PCR System (Thermo Fisher Scientific v1.4). The Protein Thermal Shift dye was diluted to 1× and mixed with 1 μg of purified protein in a final volume of 20 μL of 50 mM PBS buffer. The mixture was heated from 25 °C to 99 °C at a ramp rate of 0.05 °C/s. Fluorescence was monitored using the instrument’s default excitation/emission wavelengths for the Protein Thermal Shift dye. The apparent melting temperature (Tm) was determined from the peak of the first derivative of the resulting fluorescence melting curve using the Protein Thermal Shift software v1.4. All experiments were performed in triplicate, and data are presented as mean ± SD.

### 3.9. Molecular Dynamics Simulations

All-atom molecular dynamics simulations were performed based on the small molecule–protein complex as the initial structure using the AMBER 24 software package. For each system, hydrogen atoms were added using the LEaP module, and the system was solvated with a truncated octahedral TIP3P water box extending 10 Å from the solute. Na^+^/Cl^−^ counterions were added to neutralize the system charge. The resulting topology and parameter files were then generated for subsequent simulations.

The systems were first subjected to energy minimization, consisting of 2500 steps of steepest descent minimization followed by 2500 steps of conjugate gradient minimization. After energy minimization, the systems were heated from 0 K to 298.15 K over 200 ps under constant volume conditions with a gradual temperature ramp. Subsequently, a 500 ps NVT (constant number of particles, volume, and temperature) ensemble simulation was performed at 298.15 K to allow further equilibration of solvent molecules within the solvent box. This was followed by a 500 ps NPT (constant number of particles, pressure, and temperature) ensemble simulation for overall system equilibration. Finally, a 100 ns production simulation was carried out under the NPT ensemble with periodic boundary conditions.

### 3.10. Homology Modeling and Molecular Docking

The 3D structures of the wild-type AKR13B3–DADH fusion enzyme and the M1 mutant were predicted using AlphaFold3 (https://alphafoldserver.com/ (accessed on 21 March 2025)). AutoDock Vina v1.1.2 was used to dock the substrates into the model structures. The docking results were visualized and structural figures were generated using PyMOL3.1, with the mutated residues highlighted.

## 4. Conclusions

In this study, a semi-rational design strategy was employed. Specifically, homologous sequence-based multiple sequence alignments were generated using EVcouplings, and thermostability-associated residues were predicted using GRAPE-WEB (https://grape.wulab.xyz (accessed on 27 March 2025)). After screening single-point mutants, a combinatorial strategy was applied to construct combinatorial mutants, and their enzymatic properties were evaluated, which successfully enhanced the thermostability of the deoxynivalenol (DON)-degrading fusion enzyme AKR13B3–DADH. Consequently, a triple-point mutant, designated M1, was constructed and exhibited significantly improved thermostability compared with the WT. The half-life of M1 at 50 °C reached about 500 min, which is 16.36 times longer than that of the WT. Molecular dynamics simulations revealed that M1 adopts a more stable conformation, a more compact overall structure, and a more gradual surface exposure change than WT, which might explain the molecular mechanism underlying its enhanced thermostability. Overall, M1 not only displayed enhanced thermostability but also improved catalytic efficiency, establishing its potential as a candidate for the industrial enzymatic degradation of DON. Moreover, M1 represents a promising starting point for a second round of protein engineering.

## Data Availability

The original contributions presented in this study are included in the article/Supplementary Materials. Further inquiries can be directed to the corresponding authors.

## Supplementary Materials

The following supporting information can be downloaded at: https://www.mdpi.com/article/10.3390/ijms27177773/s1.
