# Supplementary material for: Evolutionary Adaptability Coupled with Computation-Driven Engineering for Thermostability Enhancement of a Deoxynivalenol-Detoxifying Fusion Enzyme
Source: Int J Mol Sci. 2026 Aug 30;27(17):7773. doi: 10.3390/ijms27177773 (PMC13566255; doi:10.3390/ijms27177773)
Supplement: Supplementary file 1 [file ijms-27-07773-s001.zip › ijms-4491451-supplementary.pdf]

Supplementary Materials

Evolutionary Adaptability Coupled with Computation-Driven Engineering for Thermostability Enhancement of a Deoxynivalenol-Detoxifying Fusion Enzyme

Yiting Pan, Hao Zhu, Qingwei Jiang, Bin Ma, Changhe Chen, Fengxia Lu, Huibing Chi * and Ping Zhu *

College of Food Science and Technology, Nanjing Agricultural University, Nanjing 210095, China; 2024108033@stu.njau.edu.cn (Y.P.); 2023208018@stu.njau.edu.cn (H.Z.); 2024208008@stu.njau.edu.cn (Q.J.); 2022208018@njau.edu.cn (B.M.); 9231810320@stu.njau.edu.cn (C.C.); lufengxia@njau.edu.cn (F.L.)

* Correspondence: t2023091@njau.edu.cn (H.C.); pingzhu@njau.edu.cn (P.Z.); Tel.: +86-159-5199-2713 (H.C.); +86-137-7662-7776 (P.Z.)

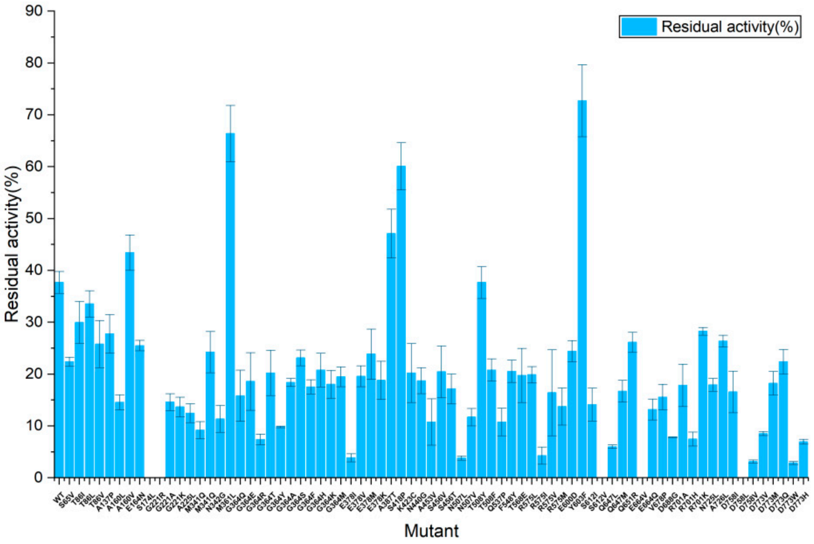

Figure S1. Residual activities of the 74 initially screened single-point mutants after heat treatment.

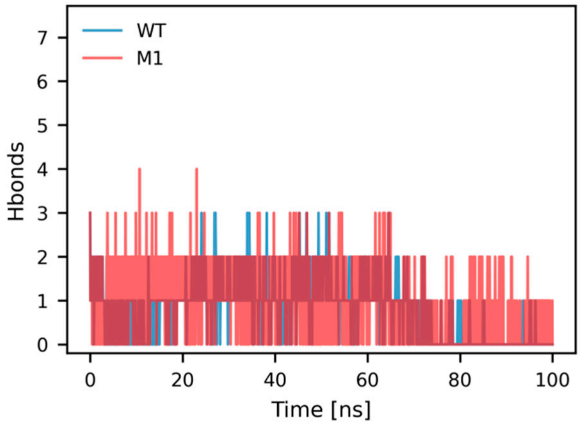

Figure S2. Number of hydrogen bonds (H-bonds) in the complex.

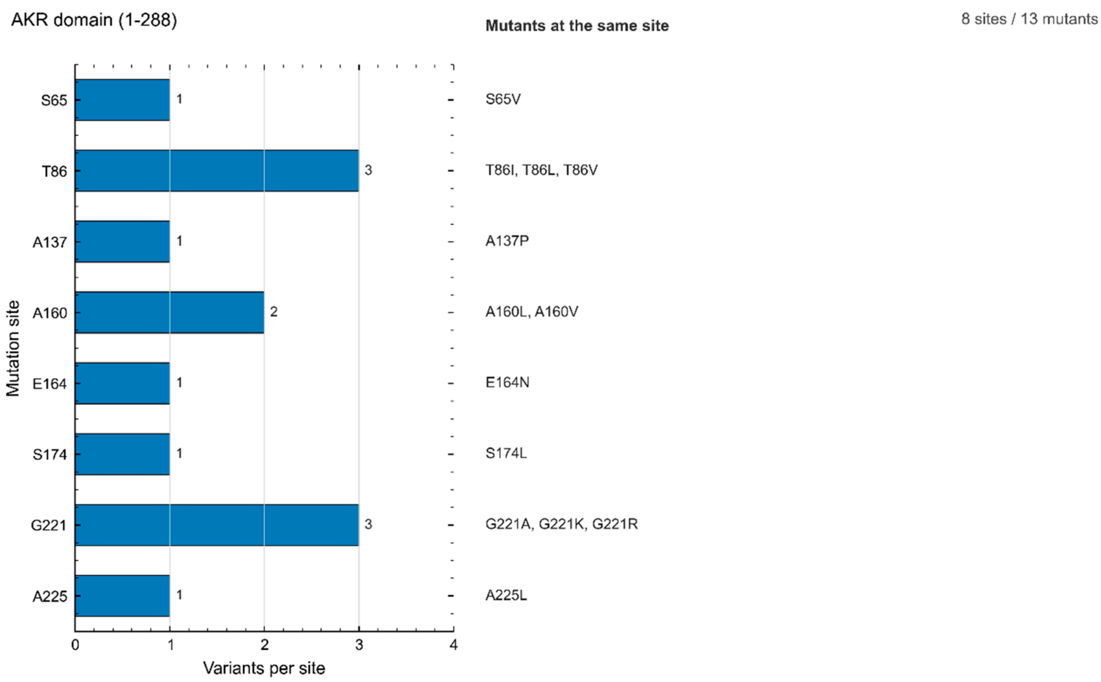

Figure S3. Distribution of the single-point mutant library across the AKR domain.

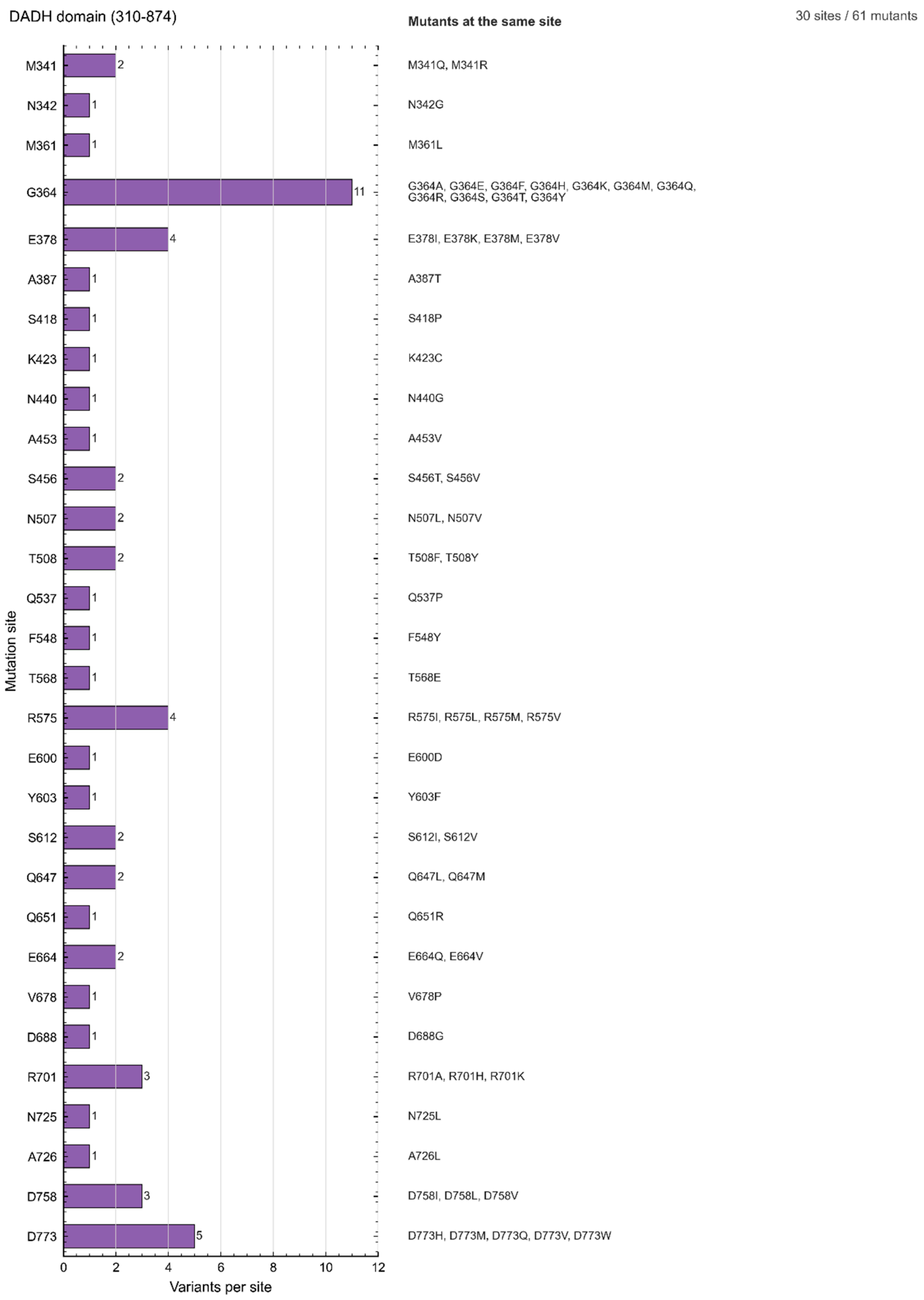

Figure S4. Distribution of the single-point mutant library across the DADH domain.

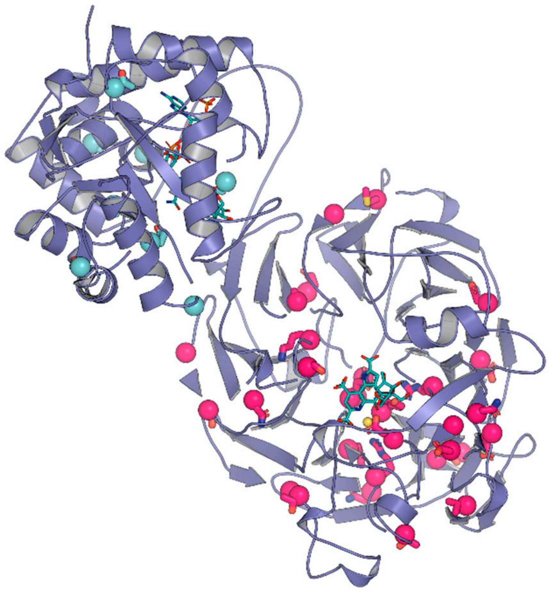

Figure S5. Structural model of AKR13B3-DADH and Screening of mutagenesis sites. Mutation sites within the DADH domain are highlighted in red, and those within the AKR domain are highlighted in cyan.

Table S1. Selected primer pairs used for site-directed mutagenesis.

Mutants	Primer
A160V-F	5'- TCGCCATgtaGGCCTTTCCGAGGTCAGCGTCG -3'
A160F-R	5'- AAAGGCCtacATGGCGAATCAGGCCTTCTTTC -3'
M361L-F	5'- AACCTGACCctcGTCTGGGGCCGCGCCCTGGA -3'
M361L-R	5'- CAGACgagGGTCAGGTTGCCGACATTGTCTTT -3'
A387T-F	5'- TGTTCATCGCCacaCCTGGCGACGTGGTGCAG -3'
S387T-R	5'- AGGtgtGGCGATGAACATTACGCCGCCGAACT -3'
S418P-F	5'- AGACACTGAACcctCTGGGTGAGAACAAGCGCG -3'
S418P-R	5'- CAGaggGTTCAGTGTCTCGCGGTCGGGCAGCG -3'
T508Y-F	5'- GCTCTGGCGCAATtatTTCATTCCGAAGGCCGGC -3'
T508Y-R	5'- AataATTGCGCCAGAGCTCTTCACCGGTAGCA -3'
Y603F-F	5'- CCtttGAAATGATCCCGGTCGACATCAACTCC -3'
Y603F-R	5'- CGGGATCATTTCaaaGGTACATTCCTGGTCCCAGTTG -3'

Table S2. Relative catalytic activities and half-lives of the wild-type and mutant AKR13B3-DADH fusion enzymes.

Mutants	Relative activity (%)	$t_{1/2}$,50°C (min)
WT	100.00	30.54±2.56
M361L	72.64±4.49	97.62±4.92
A387T	87.71±12.16	56.81±1.99
S418P	222.98±18.44	77.01±4.82
T508Y	287.18±13.06	28.41±1.02
Y603F	174.23±8.92	78.77±3.56
A160V	191.57±33.43	57.21±4.87

Table S3. Double-point combinatorial mutants.

Mutants	Site
C1	M361L-A387T
C2	M361L-S418P
C3	M361L-T508Y
C4	M361L-Y603F
C5	M361L-A160V
C6	A387T-S418P
C7	A387T-T508Y
C8	A387T-Y603F
C9	A387T-A160V
C10	S418P-T508Y
C11	S418P-Y603F
C12	S418P-A160V
C13	T508Y-Y603F
C14	T508Y-A160V
C15	Y603F-A160V

Table S4. Triple-point combinatorial mutants.

Mutants	Site
M1	T508Y/Y603F/M361L
D2	T508Y/Y603F/A387T
D3	T508Y/Y603F/S418P
D4	T508Y/Y603F/A160V

Table S5. T_m values of the wild-type and mutant M1.

Mutants	T_m (°C)
WT	50.82±0.25
M1	54.92±0.75
